# The Fascial Flap Operation for Inguinal Hernia

**Published:** 1962-01

**Authors:** Edric Wilson


### THE FASCIAL FLAP OPERATION FOR INGUINAL HERNIA

BY

EDRIC WILSON

Many surveys of the results of operation for inguinal hernia have been made, and some have shown a very high recurrence rate. The survey to be described here shows the results of one type of operation only, that described by Sir Geoffrey Keynes in 1927, and his description of its principle is worth quoting:—

"The protrusion occurs in the triangle bounded by the deep epigastric vessels on the outer side, the outer edge of the conjoint tendon on the mesial side, and the pubic bone below. The transversalis fascia is bulged through this space, and even when the wide-mouthed sac has been ligatured and removed, it is impossible to suture the conjoint tendon to the inner part of the inguinal ligament in such a way as to form a strong floor to this part of the inguinal canal; for there is an actual deficiency in the floor of the inguinal canal which must be permanently closed. A simple and satisfactory way of doing this is to use a flap derived from the internal oblique layer of the rectus sheath. The external oblique aponeurosis is lifted up, and can be separated from the internal layer without difficulty. A semi-circular flap is cut and turned over from above through 180 degrees so as to be beneath the spermatic cord. The cut edge is then sutured to the inguinal ligament from the pubic spine outwards, almost to the internal abdominal ring. The flap then completely covers the site of the protrusion and forms a new strong floor to the inguinal canal. It is not under tension and will therefore not tend to retract afterwards".

Much has been written, and many experiments made, to elucidate the cause of recurrence, but this simple explanation covers the easily ascertainable facts. Many methods, using absorbable or unabsorbable material, have been designed to cover the weak point, but their very number suggests that their success has been limited, and tissue irritation and sepsis have constantly given trouble.

Halstead (1903) recounts his experience in the treatment of hernia. His article is mainly concerned with the advantages and disadvantages of transplantation of the cord or reduction of its size, and attention is called to recurrence at the lower angle of the canal; but he mentions that four years previously, when operating on a college friend, he used a flap of "aponeurosis covering the rectus muscle, as the internal oblique was fatty and attenuated and the rectus muscle did not seem to promise so much as its fascia did". He then suggested that this method might have a wider application. The operation is illustrated by one drawing, and from this it is clear that the aponeurosis of the internal oblique, forming the deep layer of the rectus sheath, had been used as the flap. This drawing is reproduced by Jacobson (1915) with a brief description, and also by Rob and Smith (1956).

Halstead also mentions the use of a relaxation incision in the internal oblique aponeurosis (as in the Tanner slide). Bloodgood (1919) mentions Halstead's modification of transplantation of the rectus sheath and reduction in size of the cord. Edwards (1943) mentions the use of this operation for the funicular type of hernia.

Since 1936 the Keynes operation has been used for all direct or recurrent herniae, and also in indirect herniae when there is any weakness of the floor of the inguinal canal. The fascial flap covers the weak floor with living tissue which is stronger than the original floor of transversalis fascia, and is sutured without tension with a minimum amount of unabsorbable material. This is sometimes described as the Bloodgood operation, but reference to Bloodgood's (1899) description of transplantation of the rectus muscle shows that this is erroneous. I have referred to this operation as the

"Keynes fascial flap", and I think this description is justified, as Keynes (192[illegible] described the operation in detail with reasons for its use.

### THE OPERATION

The incision is extended to the level of the anterior superior spine and the extern[illegible] oblique aponeurosis is divided to the same extent. In an oblique hernia the sac [illegible] transfixed and tied at the neck with thread. If a sac is present in a direct hernia it [illegible] incised, the pro-peritoneal fat is pushed back, and the sac is closed with a purse-strin[illegible] suture. The cord is lifted up; the cremasteric vessels are divided, and the edge of th[illegible] inguinal ligament is cleared of areolar tissue from the pubic spine to the internal rin[illegible]

The fascial flap is prepared as described above in Keyne's words; some sharp dis[illegible] section is necessary as the mid-line is approached. Care must be taken to make th[illegible] flap large enough, as some rotation takes place, and in dissecting the flap off the pyra[illegible] midalis muscle care must be taken not to perforate it, for at the lateral edge of thi[illegible] muscle a fibrous septum is present and careful dissection is necessary.

When suturing the flap to the edge of the inguinal ligament, the first stitch of threa[illegible] includes the periosteum of the pubic spine. Two or three stitches are placed latera[illegible] to the cord, which is thus snugly surrounded by the flap. In placing these latera[illegible] stitches there is a tendency to approach the cut edge of the external oblique, and thi[illegible] must be guarded against. The operation can be completed in half an hour, which ha[illegible] perhaps some advantage in reducing the complications of prolonged anaesthesia.

### RESULTS OF THE OPERATION

The series to be described here is largely personal. From 1947 to 1958 inclusiv[illegible] 211 operations were performed on 186 patients, 191 by myself and 20 by registrar[illegible] There were 121 indirect and 90 direct herniae, and with this proportion of dire[illegible] herniae the series must be regarded as a somewhat unfavourable one.

It was decided to assess the results by means of a postal follow-up. The 186 patien[illegible] were written to, and asked (a) if they were completely satisfied with the operatio[illegible] (b) whether they had been able to return to their original work, and (c) whether the[illegible] was any recurrence. If in doubt, they were asked to attend for examination.

Replies were eventually received from 151 patients. Of the remaining 35, 17 we[illegible] dead, and 18 remained untraced after the help of various agencies had been enlisted t[illegible] trace patients who had left the area. However, 11 of these untraced patients woul[illegible] have been aged over 65 in 1960, and are possibly dead. At the time of the survey, th[illegible] interval that had elapsed since operation in these 151 patients is shown in the Tabl[illegible]

Time lapse since operation in 151 patients traced in 1960

| Years after operation | 2 | 3 | 4 | 5 | 6 | 7 | 8 | 9 | 10 | 11 | 12 | [illegible] |
|---|---|---|---|---|---|---|---|---|---|---|---|---|
| Number of Patients | 7 | 8 | 16 | 21 | 22 | 16 | 13 | 13 | 4 | 4 | 15 | [illegible] |

One hundred and eight of the 151 patients who were traced said that they we[illegible] satisfied with the operation, and had returned to their original work, with a few e[illegible] ceptions due to age. Many were emphatic in expressing their satisfaction and gratitud[illegible] For example, a master butcher aged 44, operated on in 1954 after two recurrence[illegible] stated that he was able to work long hours and lift heavy carcasses without discomfo[illegible]

Some justification is required for accepting these satisfactory replies as evidenc[illegible] that there had been no recurrence. It has been urged that a review which is not base[illegible] on physical examination of the patient is unsatisfactory. Shuttleworth and Davie[illegible]

(1960) found that in their own series "over 10 per cent of recurrences were unnoticed by the patient". Accepting this, it may nevertheless be held that a patient may for practical purposes be considered cured if he has not noticed a recurrence and if he feels that the disability for which he sought relief has been removed.

Only 43 patients were re-examined: 25 at their own request, either from a wish to cooperate or more usually because of some discomfort around the scar. Six recurrences were found amongst them. Ten others attended hospital for some unrelated surgical condition, and in none of these was there recurrence. Four were examined by their doctors, when, because of failure to reply, the doctor kindly examined the patient; no recurrences were found. Four who lived in Plymouth and failed to reply were visited by me, and 2 recurrences (Cases 4 and 6) were noted.

It was thus possible to examine the nature of the recurrence in 8 patients, and brief details of these cases are given below.

**Case 1, age 39.** Operation in 1951, recurred in 10 months. Re-operation 1952. Indirect sac found which had not been ligated at the neck. Much scar tissue present. Keynes operation performed. At re-examination in May 1960 there was a slight bulge at the medial end of the incision without an expansile impulse on coughing, and causing no disability.

**Case 2, age 44.** Operation by me in 1954, recorded as "modified Bloodgood". At re-operation in 1957, a direct hernia was found and there was no evidence of interference with the internal oblique layer of the rectus sheath. It is doubtful if the house surgeon's description of this operation was accurate. When re-examined in 1960 there was no recurrence.

**Case 3, age 61.** Operation in 1954 for indirect hernia, repaired by a "mersaline" darn. Recurrence in 7 months. At re-operation in 1955 a large indirect sac found; anatomy obscured by the previous darning. Keynes operation performed. Recurrence took place in 1958, and in 1960 the patient at age 67 agreed to another attempt. At this operation a direct sac was present and it was obvious that the fascial flap was too small and that the darning operation had almost destroyed the inguinal ligament. "Mersaline" was present in lengths of 3 to 4 inches. The deep layer of the rectus sheath had to some extent been re-formed, and by careful dissection it was possible to re-form a larger flap which was then rotated and sewn to the inguinal ligament. The re-formation of the deep layer of the rectus sheath in this patient was of interest. It was not of good quality, but it had shown that the fascia lata can regenerate. (In contrast, a patient who had been admitted many times under physicians for neurotic symptoms was operated on in 1953, and in 1957 claimed that the hernia had recurred. At operation in 1960 there was a firm posterior wall, and on lifting up the external oblique the rectus muscle could be plainly seen to be denuded of the internal oblique aponeurosis.)

**Case 4, age 59.** Bilateral sliding herniae, 1947, of many years duration. In 1955, after straining to lift his heavy invalid wife, he noticed a bulge on the right side. Examined in January 1961, a frail little man; bulging of the whole of the right inguinal region and a slight bulge on coughing on the left side; wears a truss.

**Case 5, age 43.** Recurrence after two operations in Canada; direct hernia. Re-operation 1954. A builder's labourer, now on lighter work and not wearing a truss. Impulse on coughing.

**Case 6, age 46.** Recurrence in 1956 after operation by me in 1954 when the sac was ligated and the internal ring tightened by thread stitches in the transversalis fascia. Now an impulse on coughing.

**Case 7, age 52.** Direct hernia; had pneumonia while convalescent (1956). Bulging of whole length of inguinal canal. It is possible that the flap had not united, and coughing had disrupted it.

**Case 8, age 52.** Indirect hernia, operation 1925. Now has a definite bulge.

Two of these recurrences can be ascribed to lapses in technique, such as failure to make an adequate flap, or inefficient suturing; and experience has shown that a badly-tied knot in thread can slip. In Cases 1, 2, and 6 the explanation may be as follows: after the flap has been sutured into position, the lateral border of the rectus muscle is exposed and the transversalis fascia in this area is sometimes covered with fat and looks weak. If, as in a large hernia, the external oblique fascia is attenuated at the external ring, then there is little support to this area. A bulge may be produced on straining, but this is not a true hernia.

### FACTORS INFLUENCING RECURRENCE

### *Age*

Cure of an indirect hernia in the young ought to be permanent in the hands of [illegible] competent surgeon. But with increasing age the abdominal musculature is degene[illegible]rating, and increasing adiposity and chronic coughs add to the risk of recurrence. Th[illegible] average age at the time of operation in this series was 51 years. The youngest patient[illegible] were two aged 20 (one of whom was operated on by a registrar for sliding hernia), on[illegible] aged 23, and one aged 29. The oldest was aged 83 at operation in 1957, alive and tend[illegible]ing his garden in 1961.

### *Type of Hernia*

Direct hernia is known to be more difficult to cure than an indirect hernia that ha[illegible] not become large enough to stretch the internal abdominal ring. In this serie[illegible] there were 121 indirect and 90 direct herniae. In the opinion of some surgeons it i[illegible] unwise to operate at the same session on both sides of bilateral hernae; the autho[illegible] does not share this opinion.

### *Sepsis*

This was common in all types of operating owing to external circumstances. [illegible] occurred in a mild form in an unknown number of the present operations, and wa[illegible] severe in a few cases, but does not appear to have influenced recurrence. A patie[illegible] aged 67, operated on for recurrent hernia in 1955 (first operation 8 years before[illegible] needed incision of a large abscess, but when re-examined in 1960 the hernia was soun[illegible] despite an almost spherical abdomen.

### *Previous Recurrence*

In a recurrent hernia the anatomy may be distorted and the tissues damaged by th[illegible] previous repair. The number of recurrent herniae originally operated upon by oth[illegible] surgeons was 21 in this series, and the interval between operation and recurren[illegible] ranged from one to ten years.

### SUMMARY

1. The results of 211 operations performed during twelve years for the repair [illegible] inguinal herniae are recorded.

2. A simple operation which follows the principles of plastic surgery is describe[illegible]

### ACKNOWLEDGEMENTS

My thanks are due to Mr. H. W. Rackham, Medical Records Officer, and his sta[illegible] for assistance in tracing the patients.

### REFERENCES

Bloodgood, J. C. (1899). *Bull. Johns. Hopk. Hosp.*, **7,** 273.
Bloodgood, J. C. (1919). *Ann. Surg.*, **52,** 81.
Edwards, H. (1943). *Brit. J. Surg.*, **31,** 122, 183.
Halstead, W. S. (1903). *Bull. Johns. Hopk. Hosp.*, **14,** 208.
Jacobson (1915). In *Operation of Surgery*, London, 1951.
Keynes, G. (1927). *Brit. med. J.* **i,** 173.
Rob, C., and Smith, R. (1956). *Operative Surgery*, London.
Shuttleworth, K. E. D., and Davies, W. H. (1960). *Lancet*, **i,** 126.

